# Genetic Evidence Supports the Multiethnic Character of Teopancazco, a Neighborhood Center of Teotihuacan, Mexico (AD 200-600)

**DOI:** 10.1371/journal.pone.0132371

**Published:** 2015-07-22

**Authors:** Brenda A. Álvarez-Sandoval, Linda R. Manzanilla, Mercedes González-Ruiz, Assumpció Malgosa, Rafael Montiel

**Affiliations:** 1 Laboratorio Nacional de Genómica para la Biodiversidad, Unidad de Genómica Avanzada, Centro de Investigación y de Estudios Avanzados del Instituto Politécnico Nacional, Irapuato, Guanajuato, Mexico; 2 Instituto de Investigaciones Antropológicas, Universidad Nacional Autónoma de México, Mexico City, Mexico; 3 Unitat d’Antropologia, Departamento de Biologia Animal, Biologia Vegetal i Ecologia, Universitat Autònoma de Barcelona, Bellaterra, Barcelona, Spain; University of Florence, ITALY

## Abstract

Multiethnicity in Teopancazco, Teotihuacan, is supported by foreign individuals found in the neighborhood center as well as by the diversity observed in funerary rituals at the site. Studies of both stable and strontium isotopes as well as paleodietary analysis, suggest that the population of Teopancazco was composed by three population groups: people from Teotihuacan, people from nearby sites (Tlaxcala-Hidalgo-Puebla), and people from afar, including the coastal plains. In an attempt to understand the genetic dynamics in Teopancazco we conducted an ancient DNA (aDNA) analysis based on mtDNA. Our results show that the level of genetic diversity is consistent with the multiethnicity phenomenon at the neighborhood center. Levels of genetic diversity at different time periods of Teopancazco’s history show that multiethnicity was evident since the beginning and lasted until the collapse of the neighborhood center. However, a PCA and a Neighbor-Joining tree suggested the presence of a genetically differentiated group (buried at the *Transitional* phase) compared to the population from the initial phase (Tlamimilolpa) as well as the population from the final phase (Xolalpan) of the history of Teopancazco. Genetic studies showed no differences in genetic diversity between males and females in the adult population of Teopancazco, this data along with ample archaeological evidence, suggest a neolocal post-marital pattern of residence in Teopancazco. Nevertheless, genetic analyses on the infant population showed that the males are significantly more heterogeneous than the females suggesting a possible differential role in cultural practices by sex in the infant sector. Regarding interpopulation analysis, we found similar indices of genetic diversity between Teopancazco and heterogeneous native groups, which support the multiethnic character of Teopancazco. Finally, our data showed a close genetic relationship between Teopancazco and populations from the “Teotihuacan corridor” and from Oaxaca and the Maya region, in agreement with previous archaeological evidence.

## Introduction

**Fig 1:**
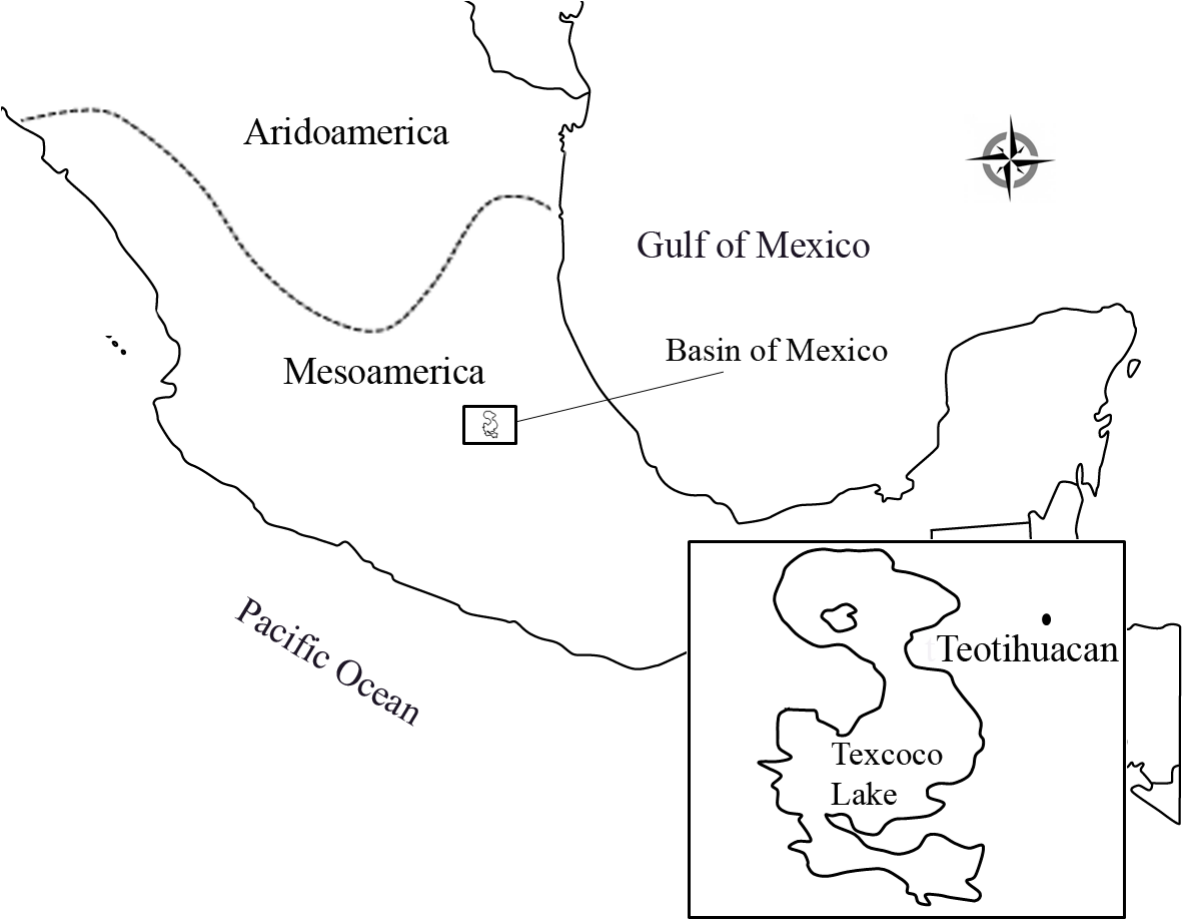
Map of Mexico in pre-Hispanic times showing the localization of Teotihuacan. The frontier between Mesoamerica and Aridoamerica is shown.

The ancient city of Teotihuacan, located in the Central Mexican highlands (Fig 1), can be considered the most influential civilization of the Classic period of Mesoamerica (AD 200–600). In the period of its greatest splendor, Teotihuacan supported a population of more than 100,000 inhabitants from different places of origin, constituting a multiethnic civilization [1, 2].

The population of Teotihuacan was organized in different social and spatial units; one of them was the multiethnic neighborhood center headed by intermediate elites [1]. Teopancazco represents one of these centers located at the southeast of the *Ciudadela* [3]. Different construction phases have been found in this site: *Tlamimilolpa* (AD 200–300) was the initial phase of the Teopancazco’s history; previous stable and strontium 87/86 isotopes studies, and a paleodietary analysis of burials from this phase [4–8] have reported evidence that the population of this phase was composed mainly of local people and of foreigners from sites belonging to the “Teotihuacan corridor to the Gulf Coast” (Puebla-Hidalgo-Tlaxcala) [9], having limited contact with other distant populations. By the end of *Tlamimilolpa* (AD 300–350), differential rituals were found which suggested the end of a constructive phase and/or drastic social changes originated by population rearrangements (*Transitional phase*) [1, 10–12]. During this time, individuals intentionally decapitated were considered foreigners, according with previous isotopic studies, coming from low altitudes and some others as foreigners coming from the “Teotihuacan corridor to the Gulf Coast” [4–9]. The latter period of the Teopacazco´s history (AD 350–550) is called *Xolalpan* (although there is also a later Metepec construction phase), and has been also characterized by previous isotopic studies [4–9] by the presence of people from the “Teotihuacan corridor” sites, from the coastal plains, reverse migrants, and local people, suggesting demographic changes during this phase in Teopancazco.

Wide archaeological evidence supporting the multiethnicity of Teopancazco has been reported: the different burial practices found in this site, the large number of faunal elements associated with the coast, foreign pottery from the Gulf Coast, cotton cloth, and foreign materials processed in Teopancazco [12, 13]. This evidence supports the idea that this neighborhood center was one articulating Teotihuacan with the Gulf Coast suggesting the presence of corridors of ally sites (i.e. the region of Puebla-Hidalgo-Tlaxcala proposed by García-Cook in 1981 [14]), through which intermediate elites sponsored caravans from Teotihuacan to sites in Veracruz, Guatemala, the “Bajío” region and Michoacán [13] in order to bring sumptuary goods and specialized labor, mainly of foreign origin [12, 13] and ultimately producing the multiethnic composition in the population of Teopancazco [1, 10, 11, 13, 15, 16–19]. Before our research, no genetic studies had been conducted in order to assess the multiethnicity of Teopancazco, characterized otherwise by ample archaeological evidence.

Regarding the population of Teopancazco characterized by sex, previous osteological analyses showed an unusual higher proportion of adult males (48.38%) in relation to adult females (ca. 15%) [9, 20]. Several studies have been conducted to detect differences in genetic diversity between adult males and females due to social organization within a population; the differences might be the result of post-marital residential patterns [21] taking into account that patrilocal pattern has been the most common residence pattern in human civilizations [22]. Spence [23] reported a biodistance analysis of cranial, dental, and postcranial traits in human remains from Teotihuacan showing a preference for patrilocality. Until now, no inferences about post-marital residence patterns present in Teopancazco have been reported. On the other hand, Gallego [24] and Alvarado-Viñas [20] reported that approximately 29% of the formal burials found in Teopancazco were infants. The study of infants also has social significance, although traditionally they have been neglected in archaeological interpretations mainly due to methodological issues [25–27]. Currently, comparisons between genetic diversity in male and female infants buried at Teopancazco have not yet been reported. These studies could help to elucidate differences in genetic diversity in the infant population according to their sex in order to find out if the differences can be explained by cultural practices, such as funerary rituals and to understand their meaning in antiquity [25].

Our genetic study from Teopancazco is the first attempt to genetically characterize the ancient population of Teopancazco assessing changes in genetic diversity through its history, from the initial period to the final construction phase of the neighborhood center, as well as to assess and compare genetic diversity by sex and by age at death of the Teopancazco individuals. Likewise, our data allowed us to assess the genetic relationships between Teopancazco and other Mesoamerican populations.

## Materials and Methods

### Sampling and Ethics Statement

The population under study came from Teopancazco, Teotihuacan, Central Mexico: Latitude North: 19.67464167, Longitude West: 98.84532778. UTM: 2, 14, Q, 516213, 2175485, “TEOPANCAZCO”. Bone and tooth samples were collected in several extensive excavation field seasons (1997–2005) and provided by Dr. Linda R. Manzanilla, head of the project “Teotihuacan. Elite and rulership. Excavations in Xalla and Teopancazco” (Last authorizations for the Project: Official letter 401.B (4)19.2013/36/0579 of the National Institute of Anthropology and History). Archaeological permits for Dr. Linda R. Manzanilla, director of the project “Teotihuacan. Elite and rulerhip”, by the Consejo de Arqueología (Archaeological Council) of the Instituto Nacional de Antropología e Historia (National Institute of Anthropology and History) are as follows: First field season: C.A. 401-36/1077 October 16, 1997. 11^th^ field season: C.A. 401-36/0787 July 11, 2003. 12th field season: C.A. 401-36/0824 July 9, 2004. The sampling process was carried out under strict criteria in order to avoid exogenous contamination. The human burial remains from Teopancazco are kept in the Instituto de Investigaciones Antropológicas (UNAM) under the custody of Dr. Linda R. Manzanilla. The samples are part of the Mexican National Heritage.

A subset of 50 samples from 46 different individuals buried at Teopancazco was sent by Dr. Linda R. Manzanilla to the aDNA laboratory in LANGEBIO, CINVESTAV, Mexico, in order to be analyzed (burials 1A, 1B, 2, 3, 4, 5, 10A, 16, 17, 35, 38, 39, 45, 46, 48, 49, 51, 55, 56, 59, 60, 61, 65, 67, 71, 78, 88, 89, 90, 91, 92, 96, 99, 100, 101, 102, 103, 105, 107, 108, 110, 111, 112, 116, RT14239, and RT12805). Additionally, a subset of eleven samples (burials 1B, 2, 3, 4, 38, 45, 49, 60, 102, 103, and 105) was sent to the Universitat Autònoma de Barcelona (UAB), Spain, in order to replicate results.

### Contamination control

DNA extraction was carried out under strict conditions for the analysis of ancient DNA, in a dedicated clean room, positively pressurized with ultra filtered and UV-irradiated air, with separation of pre- and post-PCR areas. Laboratory equipment was treated with 30% bleach for DNA contamination removal. The use of disposable filter-plugged pipette tips, tubes, protective cloths, hair covers, laboratory gloves, surgical masks, glasses, and shoe protectors to prevent contaminations was mandatory. Solutions and buffers were irradiated with ultraviolet light. Negative extraction controls and negative PCR controls were always employed.

Buccal swab samples from the personnel related to samples handling were analyzed in order to discard the possibility of contamination during excavations or laboratory procedures.

### DNA extraction

For each individual analyzed, a fragment of bone or a complete teeth, in a good state of preservation (without fissures), was used. Samples were processed in a type II B2 biological security hood located inside the clean room. The outer surface of the bone or teeth was UV-irradiated for 15 min and a dental drill was used to remove 0.1 g of powdered material. Powdered samples were added to 5 ml of extraction buffer (50 mM Tris-HCl pH 8.0, 0.425 M EDTA pH 8.0, 0.5% SDS) and 50μl of Proteinase K solution (10 mg/ml). Samples were incubated at 37°C for 24 hrs, followed by a phenol/chloroform DNA extraction [28]. Aliquots of raw DNA extracts were analyzed with a High Sensitivity DNA Assay Chip Kit on a *Bioanalyzer 2100* (Agilent) to quantify and assess the quality of extracted DNA. The result of this assay shows the distribution of DNA fragment sizes in the extract, and helps to determine the DNA fragmentation pattern of each sample. Highly degraded samples show a distribution with a peak of less than 100 bp (i.e. most of the fragments are smaller than 100 bp) [29].

Ancient DNA extraction and mtDNA amplification and sequencing in the UAB were performed as previously described [30].

### DNA amplification and Haplogroup determination

#### Amplification and cloning of an HVRI segment (positions 16190–16339)

A 149 base pair (bp) portion located between positions 16190 and 16339 (numbered according to Anderson *et al*. [31], of the D-loop region was PCR amplified with primers MT-F16190 5' CCCCATGCTTACAAGCAAGT-3' [28] and MT-R16339 5'-GTGCTATGTACGGTAAATGG-3' [32]. A 30 μL reaction contained 2 μL of aDNA extract, 1X buffer (150 mM Tris-HCl pH 8.0, 500 mM KCl), 2.5 mM MgCl_2_, 200 μM each dNTPs, 0.4 μM each primer, and 0.12 U of Bioline *Taq*DNAPolymerase (Biotaq). Thermal cycling was carried out under the following conditions: 5 minutes at 94°C; followed by 40 cycles of 0.5 minutes at 94°C, 0.5 minutes at 52°C, 0.5 minutes at 72°C; and a final step of 7 minutes at 72°C. The PCR product was then cloned using a TOPO TA Cloning kit and TOP10 competent cells (Invitrogen) following manufacturer’s instructions. A minimum of 50 colonies were selected from each cloned sample for colony PCR using the M13 primers. We then sequenced several clones and obtained between 3 and 11 clones per sample that contained the transformed vector with the fragment of interest. DNA sequencing was performed in both directions at the Genomic Services Unit in LANGEBIO, CINVESTAV. Sequences obtained were aligned to the mtDNA reference sequence (rCRS) [33] using CLC Sequence viewer v.6.5.1 (CLC bio, Aarhus, Denmark). To determine the mitochondrial haplogroup we analyzed a set of diagnostic mutations characterizing haplogroups A, B, C and D, between positions 16190–16339 [34]. The haplogroups were confirmed using HaploGrep software, which is supported by up-to-date knowledge of the mtDNA phylogeny [35], MitoMap [36] and PhyloTree website [37].

A D-loop fragment (700 bp) was amplified and sequenced in order to discard possible contamination from personnel related to samples handling.

#### Haplogroup characterization by High Resolution Melting analysis (HRM)

We developed an HRM method to determine the mitochondrial haplogroup of the ancient samples. This analysis is based on the melting temperature (Tm) difference of the amplified DNA fragments carrying diagnostic mutations located in the mitochondrial coding region belonging to Native American Haplogroups A, C, D and X. Haplogroup B was identified by the presence of a 9-pb deletion. Primers were designed to amplify short fragments (59–63 bp) and therefore the analysis was efficient even for samples for which amplification of the D-Loop fragment (149bp) was unsuccessful. Primers and conditions used are shown in Table 1. Fragments were amplified with the LightCycler 480 Real-Time PCR Instrument using the LightCycler 480 High Resolution Melting Master kit (Roche). The PCR reactions were performed in triplicate in a total volume of 20 μl containing 2 μl of template DNA, 2 mM MgCl_2_ 1X LightCycler Master-Mix (with dUTP instead of dTTP) and 0.2 μM of each primer. Thermal cycling was carried out under the following conditions: 10 minutes at 95°C; followed by 80 cycles of 30 seconds at 95°C, 30 seconds at 60°C; and a final step of 45 seconds at 72°C. After PCR the HRM analysis was performed as described [29]. The curves obtained from each haplogroup amplicon were compared with two references: one with the diagnostic mutation (positive control) and other lacking it (negative control) (Table 1).

**Table 1:** Primers used to determine Native American mitochondrial haplogroups. Diagnostic SNPs evaluated to determine each haplogroup are shown.

Haplogroup	Mutation	Primer Sequence		Tm positive control	Tm negative control
**A**	A663G	Forward	5'- CACCCCATAAACAAATAGGTT-3'	74.5	73.5
		Reverse	5'-GCATGTGTAATCTTACTAAGAGCTA-3'		
**A**	C8794T	Forward	5'-TATTGCCACAACTAACCTCCT-3'	79	80
		Reverse	5'-GTTGGGTGGTTGGTGTAAA-3'		
**B**	8281–8289 Del	Forward	5'-CCCGTATTTACCCTATAGCAC-3'	77	79
		Reverse	5'-AGTTAGCTTTACAGTGGGCTC-3'		
**C**	A13263G	Forward	5'-CAAAAAAATCGTAGCCTTCTC-3'	76.5	75
		Reverse	5'-ATGCCGATTGTAACTATTATGAG-3'		
**D**	C5178A	Forward	5'-ACGACCCTACTACTATCTCGC-3'	75.5	76.5
		Reverse	5'-TGGAATTAAGGGTGTTAGTCAT-3'		
**X**	T6221C	Forward	5'-CGCATAAACAACATAAGCTTC-3'	78.5	77.5
		Reverse	5'-CAGATGCGAGCAGGAGTAG-3'		

### Statistical analysis

#### Intrapopulation analysis

Intrapopulation statistical analyses were carried out on the haplogroup and the haplotype (sequence) level to assess if there were changes in genetic diversity in a temporal scale through the history of the neighborhood center. Teopancazco was analyzed as a whole, and with the samples divided into three different historical phases: *Tlamimilolpa* (the initial period, AD 200–300), *Transitional* (characterized by a termination ritual with decapitated males buried in the same pit, AD 300–350), and *Xolalpan* (the most active period for exchange relations, AD 350–550) [9, 12, 38].

Nei’s genetic diversity indices [39] were calculated over haplogroup (*Ĥ*a) and haplotype (*Ĥ*b) data. A one-way ANOVA was performed in order to compare Nei’s genetic diversity indices at the haplogroup level among the three chronological groups analyzed. A two-tailed t-test was performed in order to compare Nei’s gene diversities indices at the haplotype level among the temporal groups analyzed. These analyses were carried out using GraphPad Prism software v6.00 (GraphPad Software, La Jolla, CA USA, www.graphpad.com). An homogeneity test of haplogroup frequency distributions was carried out between the analyzed populations using an exact test of population differentiation [40], with 200,000 steps of the Markov chain and 5,000 dememorization steps, with α = 0.016 after Bonferroni correction. These analyses were carried out using the Arlequin software v3.5.1.2 [41].

#### Infant population analysis

The sex of the infants was previously determined by real-time PCR amplification of small fragments of the amelogenin gene, followed by High Resolution Melting analysis (HRM) [29]. Nei’s genetic diversity indices [39] were calculated over haplogroup (*Ĥ*a) data for both groups. Gene diversities were compared by a two-tailed t-test using GraphPad Prism software v6.00 (GraphPad Software, La Jolla, CA USA, www.graphpad.com). A homogeneity test of haplogroup frequency distributions was carried out by an exact test of population differentiation [40] as described above.

#### Patterns of post-marital residence

The sex of the adult individuals from Teopancazco was previously determined by osteological methods [20, 29]. Nei’s genetic diversity (*Ĥ*a) [39] was calculated over haplogroup frequencies for both female and male groups. Levels of genetic diversity were compared by a two-tailed t-test using GraphPad Prism v6.00 (GraphPad Software, La Jolla, CA USA, www.graphpad.com). A homogeneity test of haplogroup frequency distributions was carried out by an exact test of population differentiation [40] as described above. Genetic data were compared with archaeological evidence available in order to make inferences about post-marital residential pattern.

#### Interpopulation analysis

**Fig 2:**
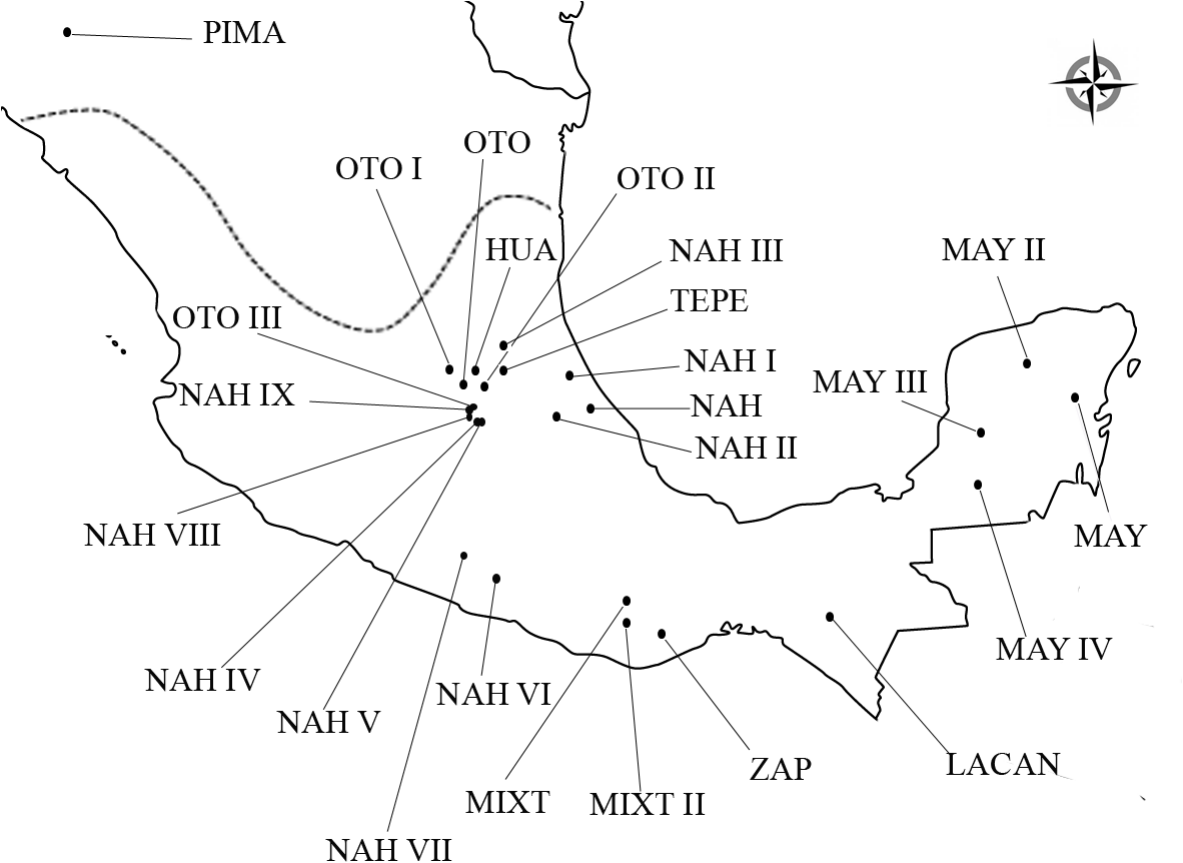
Geographical locations of indigenous American populations used in the present study.

Interpopulation statistical analyses were carried out on the haplogroup and the haplotype (sequence) level. Mitochondrial haplogroup frequencies from all reference populations are reported in S1 Table. Haplogroups frequencies from modern Mesoamerican populations located in the geographic region called “the Teotihuacan corridor to the Gulf Coast” (Hidalgo, Puebla and Veracruz), Oaxaca and Maya regions (Fig 2) [42–51] were analyzed in order to evaluate possible genetic relationships with Teopancazco. An exact test of population differentiation [40] was used as described above (α = 0.00036 after Bonferroni correction). Comparisons in levels of genetic diversity (*Ĥ*a) between Teopancazco and, Maya, Otomi, Nahua populations [44–46, 49–53] and Lacandon populations [54] were carried out by using a one-way ANOVA with a post-hoc Dunnett’s test.

A Principal Component Analysis (PCA) based on frequencies of mitochondrial haplogroups was done with XLSTAT software v2014.1.03 (Addinsoft, www.xlstat.com). A Pima population was used as external group (Aridoamerican population) due to its relatively higher genetic distance to Mesoamerican populations [51].

### Phylogenetic analysis

Haplogroup frequencies (S1 Table) were used to calculate Reynold’s genetic distances [55] between all pairs of populations analyzed, and these distances were used to construct a Neighbor-Joining tree [56]. The analysis was done using SEQBOOT, GENDIST, NEIGHBOR, and CONSENSE programs from the PHYLIP package v3.695 [57]. The tree robustness was assessed by bootstrapping (100 pseudoreplicates). A Maximum Likelihood tree was constructed from the haplotype data using the TN93 model [58]. A discrete gamma distribution was used to model evolutionary rate differences among sites (4 categories, alpha parameter = 0.9821). Nodal support was assessed by bootstrapping (1000 pseudoreplicates). Data used included the 16 sequences available from Teopancazco and sequences previously reported in GenBank [59] and HVRBase++ [60] from 9 Native Mexican populations (S2 Table). The tree was constructed with MEGA software v6.1 [61].

## Results

### Analysis efficiency and independent replication

We were able to recover mtDNA data from 29 out of the 46 available samples (63.04%). All these 29 samples amplified for short fragments (59-63pb) of the coding region, however, only 16 of them (55.17%; 34.78% from the total samples) also amplified for the 149bp fragment of the HVR-I. These 16 sequences have been deposited in GenBank (Accession numbers KR813318-KR813333). No matches were detected between sequences recovered from skeletal remains and from personnel related to samples handling (S3 Table).

We sent eleven samples (bone and teeth) to the Universitat Autònoma de Barcelona (UAB), Spain, in order to replicate results. For two of the samples (burials 2 and 3) no amplification was obtained. Nine samples were amplified; however, the sequences of five of them (burials 1B, 4, 38, 60, and 105) showed an unusual number of substitutions constituting haplotypes without phylogenetic sense and therefore were considered artifacts. Reliable sequences were obtained for only four of the eleven samples (36.36%). These sequences showed SNPs in agreement with the SNPs previously determined in the LANGEBIO laboratory for those samples (burials 45, 49, 102, and 103). The percentage of efficiency (sequences) obtained in the UAB was similar to the efficiency obtained in LANGEBIO (34.78%). This result confirms the poor DNA preservation at the site.

### Intrapopulation analysis

The haplogroup determined for each of the amplified individuals (N = 29) is reported in Table 2. All samples correspond to previously reported Native American haplogroups [62–65]. Haplogroup A is the most frequent (55%), haplogroups B and D are less represented (21% and 17% respectively), and haplogroup C has the lowest frequency (7%). Samples from Teopancazco were divided into three different temporal periods: *Tlamimilolpa* (N = 10), the *Tlamimilolpa-Xolalpan Transition* (N = 11), and *Xolalpan* (N = 8). The *Tlamimilolpa* and *Xolalpan* periods showed a similar frequency of haplogroup A (~ 60%) and B (~10%) while the frequencies in *Transitional* period were 50% and 30% respectively. Regarding haplogroup C, a twofold increase in frequency was observed in the *Tlamimilolpa* and *Transitional* periods (20%), in comparison to the *Xolalpan* phase, and, notably, the presence of haplogroup D was observed only in the *Tlamimilolpa* and *Xolalpan* phases (i.e. it is absent in the *Transitional* period) (Table 3).

**Table 2:** Burials analyzed in this study. Mitochondrial haplogroup, biological sex, and age of 29 individuals recovered from Teopancazco. The HVRI sequence from 16 of them is also shown (positions 16190–16339). Mutations are numbered according to the revised Cambridge Reference Sequence [33].

BURIAL	PHASE	HAPLOGROUP	SEX	AGE	HRM	HVRI Sequence
105 *	TLAMIMILOLPA	C	MALE	SUB ADULT	13263G	
103 *	TLAMIMILOLPA	D	FEMALE	ADULT	5178A	16223T
60 *	TLAMIMILOLPA	A	FEMALE	ADULT	663G	16223T, 16290T, 16240G
108 *	TLAMIMILOLPA	A	FEMALE	SUB ADULT	663G	16223T, 16319A
**107**	TLAMIMILOLPA	B	N.D.	INFANT	8281–8289 del	
**RT14239**	TLAMIMILOLPA	A	N.D.	N.D.	663G	
**99**	TLAMIMILOLPA	A	MALE	INFANT	663G	
**110**	TLAMIMILOLPA	C	MALE	INFANT	13263G	
**101**	TLAMIMILOLPA	A	FEMALE	INFANT	663G	
116 *	TLAMIMILOLPA	A	MALE	ADULT	663G	16229C, 16233G
**46**	TRANSITION	A	MALE	ADULT	663G	
45 *	TRANSITION	A	FEMALE	INFANT	663G	16223T, 16260T, 16290T, 16319A
**96**	TRANSITION	B	MALE	INFANT	8281–8289 del	
61 *	TRANSITION	A	FEMALE	INFANT	663G	16223T, 16290T, 16319A
89 *	TRANSITION	A	FEMALE	ADULT	663G	16223T, 16290T, 16319A
**49**	TRANSITION	B	FEMALE	INFANT	8281–8289 del	
56 *	TRANSITION	A	FEMALE	INFANT	663G	16223T, 16290T, 16319A
RT12805 *	TRANSITION	C	N.D.	N.D.	13263G	16245T
92 *	TRANSITION	C	MALE	ADULT	13263G	16227T
59 *	TRANSITION	B	MALE	INFANT	8281–8289 del	16264T, 16278T, 16311C
55 *	TRANSITION	B	MALE	ADULT	8281–8289 del	16217C
**102**	XOLALPAN	D	FEMALE	ADULT	5178A	
38 *	XOLALPAN	A	FEMALE	INFANT	663G	16223T, 16290T, 16319A
2 *	XOLALPAN	A	FEMALE	ADULT	663G	16223T, 16290T, 16319A
**3**	XOLALPAN	C	FEMALE	INFANT	13263G	
10A *	XOLALPAN	A	MALE	ADULT	663G	16223T, 16290T, 16319A
**4**	XOLALPAN	A	MALE	INFANT	663G	
**1B**	XOLALPAN	A	N.D.	ADULT	663G	
**5**	XOLALPAN	B	N.D.	ADULT	8281–8289 del	

* Samples used in Nei’s genetic diversity estimation at haplotype level reported in Table 3.

**Table 3:** Haplogroup frequencies and Nei’s genetic diversity at haplogroup (Ĥa) and haplotype (Ĥb) levels.

	*N* ^*a*^	*N* ^*b*^	A	B	C	D	*Ĥ*a	*Ĥ*b
**TEOPANCAZCO**	29	16	0.55	0.21	0.17	0.07	0.6404 ± 0.0738	0.9167 ± 0.0643
**TLAMIMILOLPA AD 200–300**	10	5	0.6	0.1	0.2	0.1	0.6444 ±0.1518	1.0000 ± 0.1265
**TRANSITIONAL AD 300–350**	11	8	0.5	0.3	0.2	0	0.6909 ± 0.0861	0.9643 ±0.0772
**XOLALPAN AD 350–550**	8	3	0.61	0.13	0.13	0.13	0.6429 ± 0.1841	N.D.

N^a^ Haplogroup sample size.

N^b^ Haplotype sample size.

N.D. Not determined.

**Fig 3:**
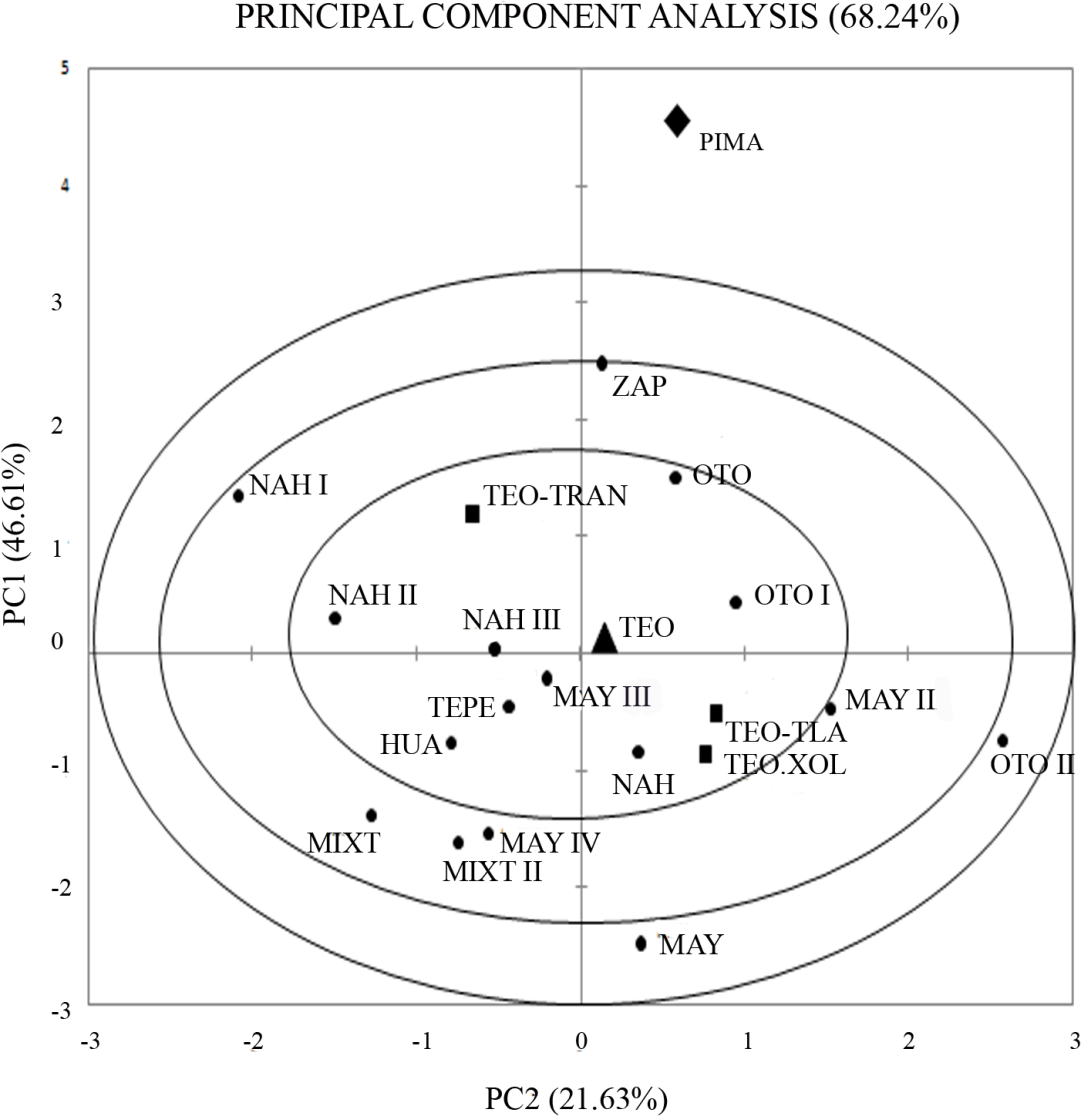
Principal Component Analysis based on mitochondrial haplogroups frequencies. **TEO** (Teopancazco), **TEO-TLAM** (*Tlamimilolpa* period), **TEO-XOL** (*Xolalpan* period), **TEO-TRAN** (*Transitional* phase), **PIMA** (Pima, Aridoamerica), **ZAP** (Zapotec, Oaxaca), **OTO** (Otomi, Hidalgo)**, OTO I** (Otomi, Hidalgo)**, OTO II** (Otomi, Hidalgo)**, NAH** (Nahua, Veracruz)**, NAH I** (Nahua, Veracruz)**, NAH II** (Nahua, Puebla), **NAH III** (Nahua, Hidalgo), **MAY** (Maya, Xcaret), **MAY II** (Maya, Yucatán), **MAY III** (Maya, Campeche), **MAY IV** (Maya, Quintana Roo), **TEPE** (Tepehua, Hidalgo), **HUA** (Huastec, Hidalgo) **MIXT** (Mixtec, Oaxaca), **MIXT II** (Mixtec, Oaxaca).

No significant differences in haplogroup frequencies among the three phases of Teopancazco were detected (global exact test of population differentiation, *p*> 0.05). However, the absence of haplogroup D in the *Transitional* period might be significant in relation to the genetic structure of this population even if not statistically significant, as discussed below. Haplogroup frequencies were used to estimate Nei’s genetic diversity for the three periods of the Teopancazco´s history (Table 3). The levels of diversity (*Ĥ*a) are similar in the three periods analyzed: *Tlamimilolpa* 0.6444 ± 0.1518, *Transitional* 0.6909 ± 0.0861, and *Xolalpan* 0.6429 ± 0.1841, and the differences were not significant (one-way ANOVA, *F* (2, 26) = 0.381, *p*> 0.05). However, the PCA (Fig 3) shows a closer proximity between *Tlamimilolpa* and *Xolalpan* groups while a differentiation of the *Transitional* period can be observed. This result is consistent with the observed topology in the Neighbor-Joining tree constructed from haplogroup frequencies. (S1 Fig).

D-loop sequences from 16 individuals buried at Teopancazco (burials 2, 10A, 38, 45, 55, 56, 59, 60, 61, 89, 92, 103, 105, 108, 116, RT12805) were used to assess haplotype diversity (Table 3) in the three historical periods. Higher levels of diversity were found in the *Tlamimilolpa* and the *Transitional* periods with no significant differences among them (two-tailed t-test, p> 0.05). Haplotype diversity in *Xolalpan* was not detected.

#### Infant population analysis

As we were able to determine sex in the infant population from Teopancazco, we analyzed genetic diversity by sex in the infants (Table 4). As in the adults, male infants showed higher genetic diversity (*Ĥ*a) than female infants (0.8000 ± 0.2086 and 0.5238 ± 0.2086, respectively), and in this case the difference between them was significant (two-tailed t-test, p< 0.05). However, no difference in haplogroup frequencies distributions was detected between both groups (exact test of population differentiation, p> 0.05).

**Table 4:** Genetic Diversity (*Ĥ*a) found in males and females (adults and infants).

POPULATION SECTOR	N	*Ĥ*a
**Female adults**	6	0.5333 ± 0.1721
**Male adults**	6	0.7333 ± 0.1552
**Female infants**	7	0.5238 ± 0.2086
**Male infants**	5	0.800 ± 0.1640

N, sample size.

#### Post-marital residential pattern

We analyzed genetic diversity by sex in a total of 12 adult individuals (Table 4). An exact test of population differentiation failed to show a significant difference in haplogroups frequencies between females and males (exact test of population differentiation, p> 0.05). We also found a higher genetic diversity (*Ĥ*a) in males (0.7333 ± 0.1552; N = 6) than in females (0.5333 ± 0.1721; N = 6) nevertheless, no significant difference in genetic diversity was found (two-tailed t-test, p> 0.05).

### Interpopulation analysis

Differences in Nei’s genetic diversity among Teopancazco, Nahua, Otomi, and Maya populations were statistically significant (one-way ANOVA, *F* (15, 680) = 385.6, *p*< 0.05). Pairwise comparisons indicated that Teopancazco shows no significant differences to one of the Maya population from Yucatán (MAY II) (Dunnett test, *p* = 0.1950), two Otomi populations (OTO I and II, Dunnett test *p* = 0.6679 and 0.8777, respectively) and three Nahua populations (NAH V, VII and IX, Dunnett test *p* = 0.6157, *p* = 0.9998 and *p* = 0.9494, respectively). Nevertheless, pairwise comparisons indicated that Teopancazco’s diversity is significantly different (*p*< 0.00036) from the diversity found in a Lacandon population, which is one of the most isolated Mexican Native group and shows a lower diversity index (0.0426 ± 0.0403).

Haplogroup frequencies were used to evaluate genetic similarities among Teopancazco and three groups of populations: the “Teotihuacan corridor to the Gulf Coast” (Hidalgo, Tlaxcala, Puebla and Veracruz), Oaxaca and Maya region. No significant differences among populations were found (global exact test of population differentiation, *p*> 0.05). This result is in agreement with the PCA (Fig 3), which shows genetic proximity between all populations, while the differentiation of the Pima (Aridoamerican population) is also evident. The topology of the Neighbor-Joining tree constructed with haplogroup frequencies from all populations also supports the observed genetic proximity between Teotihuacan (Teopancazco) and the Mesoamerican populations (S1 Fig).

Comparisons at haplotype level were assessed in order to elucidate genetic relationships among individuals from Teopancazco and from nine Native Mexican populations (S3 Table). A Maximum Likelihood tree shows that the individuals buried in Teopancazco were closer to individuals from Tepehuan, Zapotec, Maya, and Mixtec populations regardless of the period in which they were buried (S2 Fig).

## Discussion

The multidisciplinary project “Teotihuacan. Elite and rulership. Excavations in Xalla and Teopancazco”, headed by Linda R. Manzanilla, has produced relevant information supporting the multiethnicity of Teopancazco, a neighborhood center located at the southeast of Teotihuacan [9–13, 19]. However, before our research, comprehensive genetic studies aimed to better understand the multiethnicity in this site had not been carried out. In this work, we characterized the mitochondrial variability in Teopancazco, estimated levels of genetic diversity, assessed changes in genetic composition in time, analyzed the genetic diversity by sex and by age at death of Teopancazco’s individuals, and inferred the genetic relationships between Teopancazco and Mesoamerican populations.

**Fig 4:**
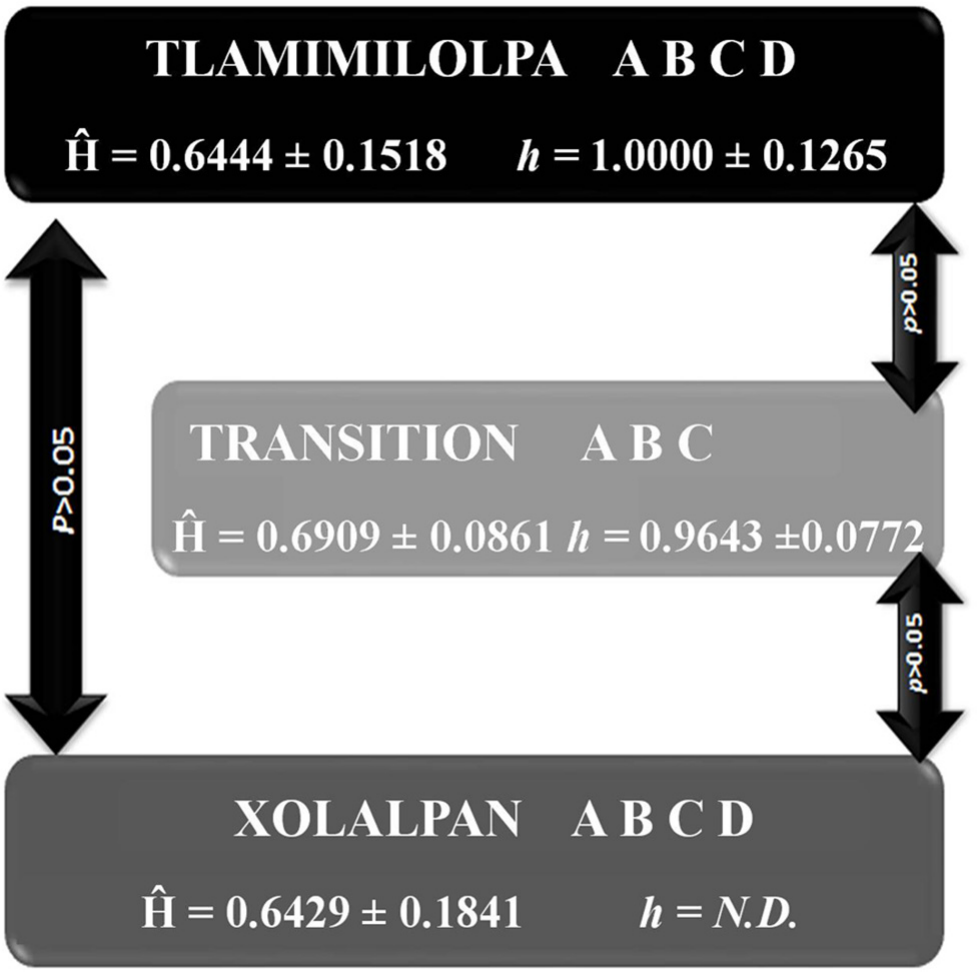
Overview of the genetic history of Teopancazco, Teotihuacan. No significant differences were observed in genetic diversity indices at haplogroup and haplotype levels between the three periods analyzed, genetic diversity at the haplotype level was estimated only for the *Tlamimilolpa* and *Transitional* periods. *P-values* of the statistical comparisons are shown inside the arrows.

Genetic analyses of the Teopancazco population from the initial to the later phases of its history allowed us to assess if there were changes in genetic diversity and composition in accordance to previous isotopic and paleodietary studies as well as archaeological evidence [4–9]. These data suggest that the population of the initial phase of Teopancazco (*Tlamimilolpa*, AD 200–350) was composed mainly by local people and by foreigners from sites belonging to the “Teotihuacan corridor to the Gulf Coast” [9]. The evidence of limited contact with other distant populations suggests a lower genetic diversity during this time in comparison to the final phase of the Teopancazco history (the *Xolalpan* phase), characterized by the possible expansion of exchange routes between Teotihuacan and Mesoamerica. However, Nei’s genetic diversity at haplogroup (*Ĥ*a) and haplotype level (*Ĥ*b) in this phase (0.644 ± 0.1518 and 1.000 ± 0.1265, respectively), was similar to the *Xolalpan* phase, suggesting that the population from Teopancazco was heterogeneous since the beginning. At the end of the *Tlamimilolpa* phase (AD 300–350), differential burial rituals were found, in which some intentionally decapitated individuals, mainly males, were considered foreigners coming from low altitudes and some others coming from the “Teotihuacan corridor to the Gulf Coast” according with previous isotopic analyses [7–9]. The increased presence of foreign people in this phase could have implied an increase in genetic diversity. As a result, we should even expect a differential genetic composition of the population of this phase in relation to the populations from the other periods analyzed. In agreement with these expectations, the haplogroup frequencies in the *Transitional* period showed a different pattern in comparison with the data observed in *Tlamimilolpa* and *Xolalpan* times. In the *Transitional* phase, the diversity at haplogroup (*Ĥ*a) and haplotype (*Ĥ*b) levels (0.690 ± 0.0861 and 0.9643 ± 0.0772, respectively) was higher than those in the initial phase in the history of Teopancazco (*Tlamimilolpa*), although the differences between both periods were not significant (*p*> 0.05). However, the absence of haplogroup D in the *Transitional* period might be indicative of a differential genetic structure of this sample, in agreement with the differential burial ritual applied to these individuals. Further evidence for the genetic differentiation of the *Transitional* group can also be observed in both the PCA plot and the Neighbor-Joining tree (Fig 3 and S1 Fig). Future studies increasing sample size or the amount of sequence information obtained might shed further light into this issue. The last part of Teopacazco´s history (the *Xolalpan* phase) was characterized, according with isotopic studies, by the presence of people from several origin sites, suggesting demographic changes caused by during this phase in Teopancazco [9]. The expansion of exchange routes in this period might have increased the genetic variability; however, we found no significant differences (*p*> 0.05) in genetic diversity (*Ĥ*a) or haplogroup frequencies between this period and the *Tlamimilolpa* or *Transitional* periods. These data suggests that the genetic diversity levels were constant from the initial phases of Teopancazco until the last part of the neighborhood history (Fig 4), and that the possible increase of trade with foreign populations previously proposed had no effect in mitochondrial genetic variability of Teopancazco’s population. The concurrent presence of haplogroup D also reinforces the idea of genetic continuity at the haplogroup level between *Tlamimilolpa* and *Xolalpan* times, contrasting with the discontinuity observed in the *Transitional* group, which lacks haplogroup D and that is separated from *Tlamimilolpa* and *Xolalpan* in both the PCA plot and the Neighbor-Joining tree, as mentioned above. Regarding the haplotype data, we found high levels of genetic diversity in the *Tlamimilolpa* and *Transitional* phases with no significant differences between them (*p*> 0.05). These data also support the multiethnic character of Teopancazco. Genetic diversity at haplotype level in *Xolalpan* was not determined because the three haplotypes obtained were identical.

Previous studies had reported that approximately 29% of the formal burials found in Teopancazco were infants [9], and no genetic data about them have been reported. Thus, our analysis of genetic sex determination represents the first attempt to analyze this population sector. After the analysis of 16 infant individuals, we found an equal proportion of male and females [29]. Genetic information from the infant sector can help to understand the role that infants played in ceremonial events within a population (i.e. human sacrifices), and in particular to infer if there were differential burial pattern between sexes. For this reason, we compared, for the first time, genetic diversity indices (*Ĥ*a) between infant males and females in Teopancazco. Our results indicated that there is a significantly higher genetic diversity in male infants compared to female infants (0.8000 ± 0.2086 and 0.5238 ± 0.2086, respectively; *p*< 0.05). This means the female group was more homogeneous than the male group, as observed for the adult population (described above). In order to find out if the differences in genetic diversity between sexes can be explained by cultural practices, such as funerary rituals, we then analyzed when and where the infants were buried. In the *Transitional* period, six perinatal individuals were placed in a flexed position on top of a large pit (surrounded by other small pits) [10] with adult heads (mainly male) each inside a vessel. Sex determination showed that four of these perinatal individuals were females (burials 45, 49, 56, and 61), while one was male and the other one was not sampled. The females were set to the western corners of the pit, while the male and the infant of undetermined sex to the east. This evidence suggests a possible relation between burial orientation and sex in infants found in this specific termination ritual. Genetic data suggest that females buried at this ritual belonged to a genetically homogeneous group. Three of the infant females of the termination ritual (burials 45, 56, and 61) belonged to mitochondrial haplogroup A, while the last one (burial 49) belonged to haplogroup B. Sequence data indicated no relationships at haplotypic level between the females. This is the first time that hypotheses based on genetic data in relation to infant sacrifices in Mesoamerican populations are proposed, and warrants further investigations.

Teopancazco was a neighborhood center characterized by an unusual higher proportion of adult males (48.38%) in relation to adult females (10–15%) [9, 20]. Before our research, no genetic data had been addressed in order to compare genetic diversity between males and females and elucidate the patterns of post-marital residence of people buried in this neighborhood center. Thus, we analyzed genetic diversity in individuals grouped by sex, in groups of similar sample size and we compared genetic data with archaeological evidence in order to suggest a possible post-marital residence pattern. Nei’s genetic diversity (*Ĥ*a) was higher in males than in females (0.7333 ± 0.1552 and 0.5333 ± 0.1721, respectively); however, this difference between them was not significant (p> 0.05). Likewise, the two groups were not different in haplogroup frequencies (*p*> 0.05). These data suggest that females were as heterogeneous as males, which is a characteristic of a neolocal pattern of post-marital residence, the establishment of a new couple in an independent place of residence away from the relatives of either spouse [66]. Sample sizes are low (see Table 3) and a lack of power in the statistical test could explain the lack of significance in the observed differences in genetic diversity. However, in a neolocal population, foreign people with high degrees of mobility are correlated with exchange systems [66–68], a characteristic observed in Teopancazco, especially in the *Xolalpan* phase. In addition, it has been proposed that when an elite controls resources and property, the remaining population follows a pattern of neolocal residence [69, 70], and it has been also proposed that an intermediate elite ruled Teopancazco as well as other neighborhood centers in Teotihuacan [10, 12, 18]. Furthermore, the neolocal hypothesis may be accurate only if we can assume that the adults married in Teopancazco and the couple was also buried in Teopancazco, and the different non-Teotihuacan burials belonging to females and males found in Teopancazco supports this assumption (i.e. burial 102, a ritualized burial from a foreigner as indicated by the isotopic analysis). Thus, Teopancazco had many of the characteristics that could have promoted a neolocal population, contrary to the patrilocality pattern proposed by Spence [23].

Levels of mitochondrial genetic diversity found in Teopancazco, at haplogroup (0.6404 ± 0.0738) and haplotype level (0.9167 ± 0.0643), supported the heterogeneous character of the site, previously proposed based on evidence indicating trade and migration with different areas and populations. Our genetic data shows genetic diversity levels similar to populations that can be considered heterogeneous due to their high genetic fluxes with Mesoamerican populations [51–52, 71].

Archaeological evidence suggests strong relationships between Teopancazco and several Mesoamerican regions, mainly with ally sites towards the coastal region of the Gulf of Mexico through the presence of a “Teotihuacan corridor” (Tlaxcala-Hidalgo-Puebla) originally suggested by García-Cook [14]. These relationships are represented by the presence of similar architecture elements, burial rituals, and pottery similar to Teotihuacan’s [72–74]. Likewise, previous isotopic analyses reported the presence in Teopancazco of possible migrants from Oaxaca, Puebla, Hidalgo and Veracruz [7, 8]. A possible relationship with Maya populations was based on information about a possible long-distance gene flow originated by bride exchange, practiced between Teotihuacan and Mayan elites [75], as well as trading relationships among Maya region and populations from Central Mexico via the Gulf Coast [76–77]. In order to elucidate if trade connections were reflected in genetic relationships, we compared Teopancazco with populations located in the “Teotihuacan corridor to the Gulf Coast”, Oaxaca, Veracruz and the Maya regions. Our genetic data show a genetic homogeneity between Teopancazco and all populations analyzed (global exact test of population differentiation, *p*> 0.05), represented also in a PCA plot (Fig 3) which shows a clear close relationship among all the Mesoamerican populations included, in agreement with the genetic homogeneity reported previously in Mesoamerica [51]. The closer genetic relationships were found between Teopancazco and populations from the “Teotihuacan corridor” (OTO, OTO I, HUA, TEPE, NAH II and III) and populations from Veracruz (NAH), in agreement with the idea that this region should be related to Teopancazco in geographical and trade terms. Maya populations from Quintana Roo (MAY IV) and Yucatán (MAY III); a Nahua population from Veracruz (NAH I), and Zapotec and Mixtec populations from Oaxaca (ZAP and MIXT and MIXT II) were the second group of populations more genetically related to Teopancazco in agreement with the increase of geographical distance from Teotihuacan. On the contrary, the more distant genetic relationships were found between Teopancazco and the Maya population from Xcaret (MAY), an Otomi population from a mountain region of Hidalgo (OTO II), and a Pima population (PIMA), suggesting that these populations were the most isolated in genetic and geographical terms from Teopancazco. It is noteworthy that a Maya population from Campeche (MAYIII) was genetically the most closely related to Teopancazco, even more than populations from the corridor. However, no presence of Mayan elements in Teopancazco has been reported. This pattern of relationships can also be observed in a Neighbor-Joining tree based on haplogroup frequencies (S1 Fig) and in a Maximum Likelihood tree based on haplotype data (S2 Fig), which shows the genetic proximity of Teopancazco to Maya, Tepehua, Mixtec, and Zapotec populations.

Our results show that the genetic relationships between Teopancazco and populations from the corridor are closer than the relationships with Maya and Oaxaca regions, in agreement with the idea that most of the emigrants from Teotihuacan did not travel very far [78]. Our results are also in agreement with the idea that the Teotihuacan state expanded throughout the Basin of Mexico, creating a large sustaining hinterland for the support of its large urban center, by means of trade, political and possible human networks [12] that could imply an effect in the population’s genetic diversity and could be the main reason for the multiethnic character of Teopancazco.

In summary, our results imply that the population of Teopancazco was heterogeneous at mtDNA level since the initial phase of the history of Teopancazco until its abandonment, although the presence of a possible genetically differentiated group, buried during the *Transitional* phase between *Tlamimilolpa* and *Xolalpan* has been detected, in agreement with the peculiar way in which they were ritualized. Regarding the post-marital residence pattern in Teopancazco, our genetic data, compared with archaeological evidence, suggest the presence of a neolocal pattern. Genetic analysis of the infant population from Teopancazco allowed us to put forward hypotheses about the termination ritual found in the *Transitional* period. We found similar indices of genetic diversity between Teopancazco and other heterogeneous civilizations such as Maya, Otomi and Nahua, which support the multiethnic character of Teopancazco. Our data also suggest a close genetic relationship between Teopancazco and populations from the “Teotihuacan corridor to the Gulf Coast” and from Oaxaca, in agreement with previous archaeological evidence.

## Supporting Information

S1 Fig Neighbor-Joining tree using Reynold´s genetic distances calculated between all pairs of populations analyzed using haplogroups frequencies. **TEO** (Teopancazco), **TEO-TLAM** (*Tlamimilolpa* period), **TEO-XOL** (*Xolalpan* period), **TEO-TRAN** (*Transitional* phase), **PIMA** (Pima, Aridoamerica), **ZAP** (Zapotec, Oaxaca), **OTO** (Otomi, Hidalgo)**, OTO I** (Otomi, Hidalgo)**, OTO II** (Otomi, Hidalgo)**, NAH** (Nahua, Veracruz)**, NAH I** (Nahua, Veracruz)**, NAH II** (Nahua, Puebla), **NAH III** (Nahua, Hidalgo), **MAY** (Maya, Xcaret), **MAY II** (Maya, Yucatán), **MAY III** (Maya, Campeche), **MAY IV** (Maya, Quintana Roo), **TEPE** (Tepehua, Hidalgo), **HUA** (Huastec, Hidalgo) **MIXT** (Mixtec, Oaxaca), **MIXT II** (Mixtec, Oaxaca).
(TIF)

S2 Fig Maximum Likelihood tree based on haplotype data from individuals recovered from Teopancazco and native Mexican populations. (TIF)

S1 Table Mitochondrial haplogroup frequencies from all reference populations used in the interpopulation analyses. * Ancient DNA Studies.
(DOCX)

S2 Table Mitochondrial haplotypes from the Mexican populations used in this study. (DOCX)

S3 Table Mitochondrial haplotype from personnel related to samples handling. (DOCX)
