# Supplementary figures and images for: Genetic Evidence Supports the Multiethnic Character of Teopancazco, a Neighborhood Center of Teotihuacan, Mexico (AD 200-600)
Source: PLoS One. 2015 Jul 22;10(7):e0132371. doi: 10.1371/journal.pone.0132371 (PMC4511806; doi:10.1371/journal.pone.0132371)

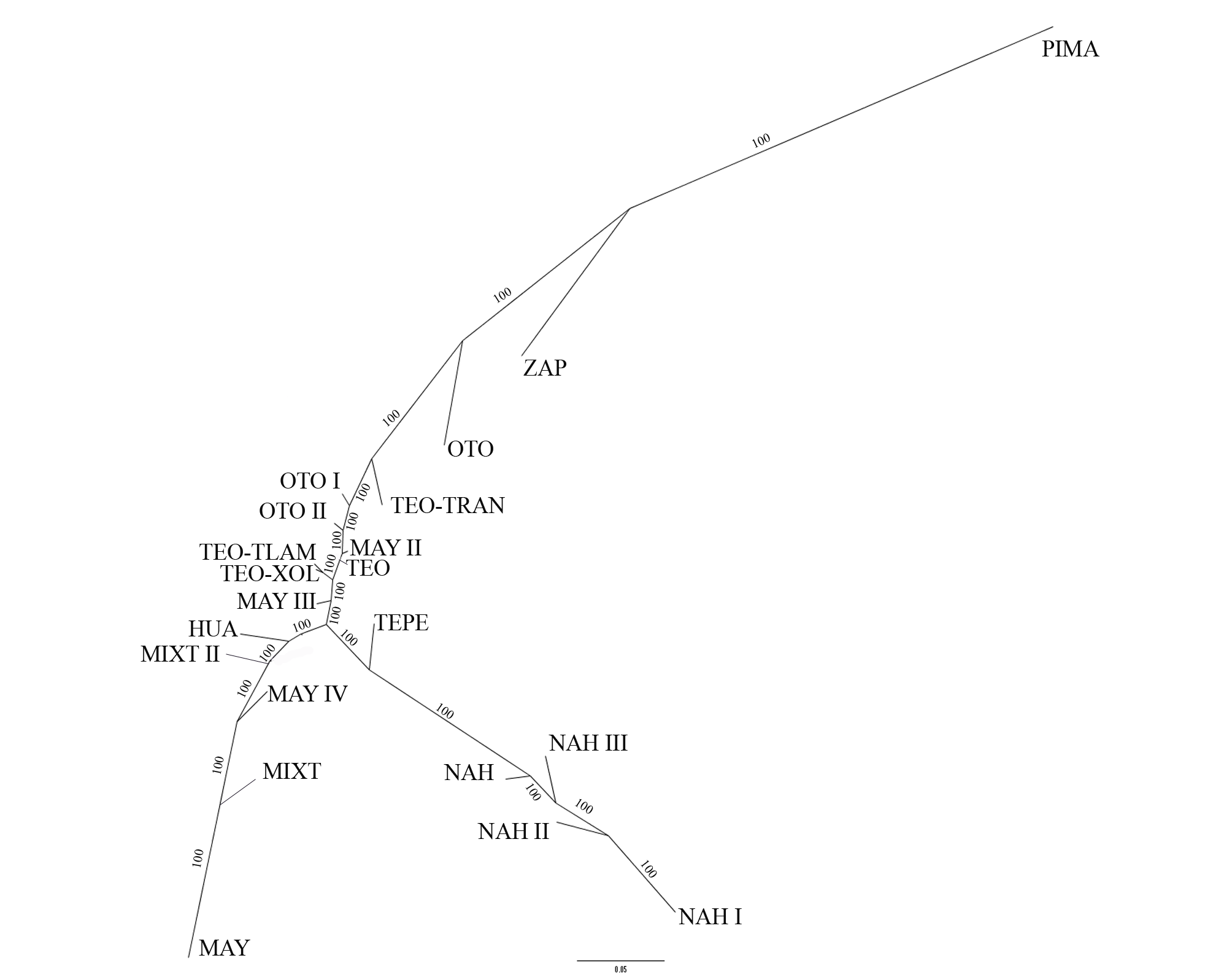

Supplement: S1 Fig — TEO (Teopancazco), TEO-TLAM (Tlamimilolpa period), TEO-XOL (Xolalpan period), TEO-TRAN (Transitional phase), PIMA (Pima, Aridoamerica), ZAP (Zapotec, Oaxaca), OTO (Otomi, Hidalgo), OTO I (Otomi, Hidalgo), OTO II (Otomi, Hidalgo), NAH (Nahua, Veracruz), NAH I (Nahua, Veracruz), NAH II (Nahua, Puebla), NAH III (Nahua, Hidalgo), MAY (Maya, Xcaret), MAY II (Maya, Yucatán), MAY III (Maya, Campeche), MAY IV (Maya, Quintana Roo), TEPE (Tepehua, Hidalgo), HUA (Huastec, Hidalgo) MIXT (Mixtec, Oaxaca), MIXT II (Mixtec, Oaxaca). (TIF) [file pone.0132371.s001.tif]

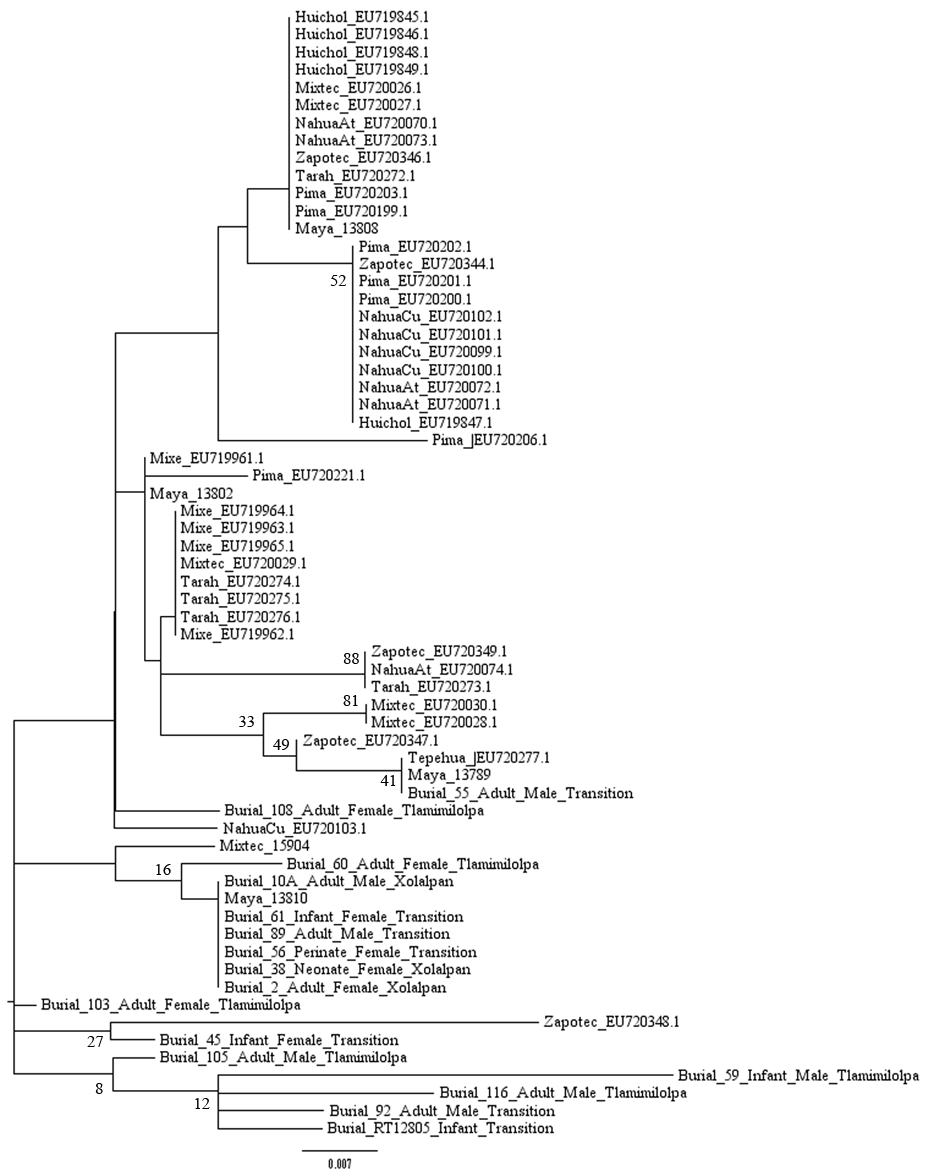

Supplement: S2 Fig — (TIF) [file pone.0132371.s002.tif]
