# Supplementary material for: Genetic Evidence Supports the Multiethnic Character of Teopancazco, a Neighborhood Center of Teotihuacan, Mexico (AD 200-600)
Source: PLoS One. 2015 Jul 22;10(7):e0132371. doi: 10.1371/journal.pone.0132371 (PMC4511806; doi:10.1371/journal.pone.0132371)
Supplement: S1 Table — * Ancient DNA Studies. (DOCX) [file pone.0132371.s003.docx]

| POPULATION | CODE | N | A | B | C | D | OTHER | REFERENCE |
| --- | --- | --- | --- | --- | --- | --- | --- | --- |
| TEOTIHUACAN (Teopancazco)* | TEO | 25 | 0.64 | 0.16 | 0.12 | 0.08 | 0 | This study |
| ZAPOTEC (Oaxaca) | ZAP | 15 | 0.33 | 0.33 | 0.33 | 0 | 0 | [42] |
| PIMA (Sonora) | PIMA | 30 | 0.06 | 0.5 | 0.43 | 0 | 0 | [42] |
| MIXTEC (Oaxaca) | MIXT | 29 | 0.82 | 0.1 | 0.07 | 0 | 0 | [42, 43] |
| MAYA (Yucatán) | MAY II | 27 | 0.52 | 0.22 | 0.14 | 0.07 | 0.03 | [44, 45] |
| NAHUA (Puebla) | NAH II | 31 | 0.61 | 0.32 | 0.06 | 0 | 0 | [43, 46] |
| MAYA (Xcaret)* | MAY | 25 | 0.88 | 0.04 | 0.08 | 0 | 0.04 | [47] |
| HUASTEC (Hidalgo) | HUA | 97 | 0.67 | 0.21 | 0.04 | 0.06 | 0 | [48] |
| NAHUA (Ixhuatlancillo, Veracruz) | NAH | 47 | 0.55 | 0.27 | 0 | 0.17 | 0 | [49] |
| NAHUA (Mexico City.) | NAH V | 59 | 0.47 | 0.35 | 0.11 | 0.05 | 0 | [49] |
| NAHUA (Chilacachapa, Guerrero) | NAH VII | 41 | 0.46 | 0.34 | 0.07 | 0.12 | 0 | [49] |
| NAHUA (Zitlala, Guerrero) | NAH VI | 46 | 0.65 | 0.3 | 0.02 | 0.02 | 0 | [49] |
| MIXTEC (Oaxaca) | MIXT II | 19 | 0.79 | 0.1 | 0.05 | 0.05 | 0 | [50] |
| MAYA (Campeche) | MAY III | 52 | 0.61 | 0.17 | 0.15 | 0.05 | 0 | [50] |
| OTOMI (Hidalgo) | OTO | 68 | 0.39 | 0.25 | 0.29 | 0.05 | 0 | [50] |
| NAHUA (Xochimilco) | NAH IV | 35 | 0.77 | 0.14 | 0.08 | 0 | 0 | [50] |
| NAHUA (Necoxtla, Veracruz) | NAH I | 25 | 0.48 | 0.52 | 0 | 0 | 0 | [50] |
| MAYA (Quintana Roo) | MAY IV | 44 | 0.79 | 0.06 | 0.09 | 0.04 | 0 | [51] |
| OTOMI VALLEY (Hidalgo) | OTO I | 81 | 0.49 | 0.14 | 0.27 | 0.08 | 0 | [51] |
| OTOMI MONTAIN (Hidalgo) | OTO II | 94 | 0.52 | 0.1 | 0.23 | 0.1 | 0.03 | [51] |
| TEPEHUA (Hidalgo) | TEPE | 54 | 0.62 | 0.25 | 0.05 | 0.03 | 0.01 | [51] |
| NAHUA (Hidalgo) | NAH III | 192 | 0.57 | 0.28 | 0.09 | 0.04 | 0.005 | [51] |
| NAHUA (Tlatelolco)* | NAH VIII | 23 | 0.65 | 0.13 | 0.04 | 0.17 | 0 | [52] |
| OTOMÌ (Xaltocan)* | OTO  III | 10 | 0.3 | 0.3 | 0 | 0.4 | 0 | [53] |
| NAHUA (Xaltocan)* | NAH  IX | 15 | 0.6 | 0.2 | 0.06 | 0.13 | 0 | [53] |
| LACANDONA (Chiapas) | LACAN | 47 | 0.98 | 0 | 0.02 | 0 | 0 | [54] |
