# Supplementary material for: Genetic Evidence Supports the Multiethnic Character of Teopancazco, a Neighborhood Center of Teotihuacan, Mexico (AD 200-600)
Source: PLoS One. 2015 Jul 22;10(7):e0132371. doi: 10.1371/journal.pone.0132371 (PMC4511806; doi:10.1371/journal.pone.0132371)
Supplement: S2 Table — (DOCX) [file pone.0132371.s004.docx]

| POPULATION | ID | NAME | DATABASE |
| --- | --- | --- | --- |
| Zapotec | EU720349.1 | Zapotec 066 | GenBank |
| Zapotec | EU720348.1 | Zapotec 062 | GenBank |
| Zapotec | EU720347.1 | Zapotec034 | GenBank |
| Zapotec | EU720346.1 | Zapotec073 | GenBank |
| Zapotec | EU720344.1 | Zapotec069 | GenBank |
| Tepehua | EU720277.1 | Tepehuan01 | GenBank |
| Tarahumara | EU720276.1 | Tarah094 | GenBank |
| Tarahumara | EU720275.1 | Tarah084 | GenBank |
| Tarahumara | EU720274.1 | Tarah077 | GenBank |
| Tarahumara | EU720273.1 | Tarah072 | GenBank |
| Tarahumara | EU720272.1 | Tarah082 | GenBank |
| Pima | EU720221.1 | Pima124 | GenBank |
| Pima | EU720206.1 | Pima121 | GenBank |
| Pima | EU720203.1 | Pima108 | GenBank |
| Pima | EU720202.1 | Pima103 | GenBank |
| Pima | EU720201.1 | Pima101 | GenBank |
| Pima | EU720200.1 | Pima099 | GenBank |
| Pima | EU720199.1 | Pima089 | GenBank |
| Nahua | EU720103.1 | NahuaCu057 | GenBank |
| Nahua | EU720102.1 | NahuaCu039 | GenBank |
| Nahua | EU720101.1 | NahuaCu037 | GenBank |
| Nahua | EU720099.1 | NahuaCu026 | GenBank |
| Nahua | EU720100.1 | NahuaCu036 | GenBank |
| Nahua | EU720074.1 | NahuaAt68 | GenBank |
| Nahua | EU720073.1 | NahuaAt64 | GenBank |
| Nahua | EU720072.1 | NahuaAt63 | GenBank |
| Nahua | EU720071.1 | NahuaAt42 | GenBank |
| Nahua | EU720070.1 | NahuaAt39 | GenBank |
| Mixtec | EU720030.1 | Mixtec063 | GenBank |
| Mixtec | EU720029.1 | Mixtec050 | GenBank |
| Mixtec | EU720028.1 | Mixtec042 | GenBank |
| Mixtec | EU720027.1 | Mixtec048 | GenBank |
| Mixtec | EU720026.1 | Mixtec046 | GenBank |
| Mixe | EU719965.1 | Mixe051 | GenBank |
| Mixe | EU719964.1 | Mixe046 | GenBank |
| Mixe | EU719963.1 | Mixe033 | GenBank |
| Mixe | EU719962.1 | Mixe028 | GenBank |
| Mixe | EU719961.1 | Mixe019 | GenBank |
| Huichol | EU719849.1 | Huichol62 | GenBank |
| Huichol | EU719848.1 | Huichol61 | GenBank |
| Huichol | EU719847.1 | Huichol58 | GenBank |
| Huichol | EU719846.1 | Huichol54 | GenBank |
| Huichol | EU719845.1 | Huichol53 | GenBank |
| Maya | 13789 | Maya92 | HVRBase++ |
| Maya | 13802 | Maya93 | HVRBase++ |
| Maya | 13808 | Maya94 | HVRBase++ |
| Maya | 13810 | Maya95 | HVRBase++ |
| Mixtec | 15904 | Mixtec | HVRBase++ |
