# Supplementary material for: Genetic Evidence Supports the Multiethnic Character of Teopancazco, a Neighborhood Center of Teotihuacan, Mexico (AD 200-600)
Source: PLoS One. 2015 Jul 22;10(7):e0132371. doi: 10.1371/journal.pone.0132371 (PMC4511806; doi:10.1371/journal.pone.0132371)
Supplement: S3 Table — (DOCX) [file pone.0132371.s005.docx]

|  | ***Mitochodrial haplotype (16190-16339)*** |
| --- | --- |
| ***Individual 1*** | ***rCRS*** |
| ***Individual 2*** | ***16223T, 16290T, 16319A, 16325C*** |
| ***Individual 3*** | ***16224C*** |
| ***Individual 4*** | ***rCRS*** |
| ***Individual 5*** | ***16298C*** |
